# The prevalence of *Campylobacter* spp., *Listeria monocytogenes* and Shiga toxin‐producing *Escherichia coli* in Norwegian dairy cattle farms: A comparison between free stall and tie stall housing systems

**DOI:** 10.1111/jam.15512

**Published:** 2022-03-11

**Authors:** Lene Idland, Erik G. Granquist, Marina Aspholm, Toril Lindbäck

**Affiliations:** ^1^ Department of Paraclinical Sciences, Faculty of Veterinary Medicine Norwegian University of Life Sciences Ås Norway; ^2^ Department of Production Animal Clinical Sciences, Faculty of Veterinary Medicine Norwegian University of Life Sciences Ås Norway

**Keywords:** Agriculture, *Campylobacter*, Food safety, *Listeria*, STEC (Shiga toxin‐producing *E. coli*)

## Abstract

**Aims:**

This study explored how dairy farm operating systems with free‐stall or tie‐stall housing and cow hygiene score influence the occurrence of zoonotic bacteria in raw milk.

**Methods and Results:**

Samples from bulk tank milk (BTM), milk filters, faeces, feed, teats and teat milk were collected from 11 farms with loose housing and seven farms with tie‐stall housing every second month over a period of 11 months and analysed for the presence of STEC by culturing combined with polymerase chain reaction and for *Campylobacter* spp. and *L. monocytogenes* by culturing only. *Campylobacter* spp., *L. monocytogenes* and STEC were present in samples from the farm environment and were also detected in 4%, 13% and 7% of the milk filters, respectively, and in 3%, 0% and 1% of BTM samples. Four STEC isolates carried the *eae* gene, which is linked to the capacity to cause severe human disease. *L. monocytogenes* were detected more frequently in loose housing herds compared with tie‐stalled herds in faeces (*p* = 0.02) and feed (*p* = 0.03), and *Campylobacter* spp. were detected more frequently in loose housing herds in faeces (*p* < 0.01) and teat swabs (*p* = 0.03). An association between cow hygiene score and detection of *Campylobacter* spp. in teat milk was observed (*p* = 0.03).

**Conclusion:**

Since some samples collected from loose housing systems revealed a significantly higher (*p* < 0.05) content of *L. monocytogenes* and *Campylobacter* spp. than samples collected from tie‐stalled herds, the current study suggests that the type of housing system may influence the food safety of raw milk.

**Significance and Impact of the Study:**

This study highlights that zoonotic bacteria can be present in raw milk independent of hygienic conditions at the farm and what housing system is used. Altogether, this study provides important knowledge for evaluating the risk of drinking unpasteurized milk.

## INTRODUCTION

Pasteurization of cow milk has been a practice in Europe since the 1880s to protect consumers from microbial pathogens (Steele, 2000). Serious human diseases such as tuberculosis, brucellosis and diphtheria have dramatically decreased with the introduction of industrial methods for thermal processing of milk (Lucey, 2015). As it poses a risk to public health, commercial distribution of unpasteurized milk (UPM) is legally restricted in the European Union (EU) (Regulation [EC] No 853/2004). However, since the beginning of the 21st century, consumption of UPM has grown in popularity in the Western world (Alegbeleye et al., 2018). This trend is based on the belief that UPM tastes better, has probiotic effects and is more nutritious compared with its pasteurized counterpart (Claeys et al., 2013; Crotta et al., 2016). However, there is sparse scientific evidence that support these claims. To meet consumers demands, some farmers and other sectors in the agricultural community in Norway have requested relaxed rules for selling UPM (Jørgensen et al., 2005).

Cattle can be asymptomatic carriers of *Campylobacter, L. monocytogenes,* and Shiga toxin producing *E. coli* (STEC) and shed the pathogens to the farm environment via their faeces. From the environment, the pathogens can spread further to the udders, milking utensils, filters and bulk storage vessels if washing and cleaning procedures are improper leading to raw milk contamination (Chlebicz & Śliżewska, 2018; Roberts & Wiedmann, 2003; Sapountzis et al., 2020). Other studies and reports highlight these bacteria as hazards related to consumption of UPM (Artursson et al., 2018; Castro et al., 2017; De Buyser et al., 2001; Jaakkonen et al., 2019; Langer et al., 2012; Lundén et al., 2004). *Campylobacter* is the most frequently reported cause of food poisoning in Europe (European Food Safety Authority and European Centre for Disease Prevention and Control, 2016), and isolates from dairy farms show genetic similarity to isolates from human campylobacteriosis cases (An et al., 2018). *Listeria monocytogenes* causes the food‐borne disease listeriosis, especially in elderly, pregnant women, infants and people with weakened immune systems (Ricci et al., 2018). Some STECs can cause foodborne disease with symptoms ranging from uncomplicated diarrhoea to bloody diarrhoea and haemolytic uremic syndrome (HUS). The World Health Organization (WHO) has estimated that 10% of patients with STEC infections develop HUS with a mortality rate of 2%–5%. The Shiga toxin (Stx) is the major virulence factor of STEC for which encoding genes are carried by bacteriophages (Stx phages) (Łoś et al., 2011). The adhesin; Intimin, encoded by *eae*, is another important virulence factor of STEC involved in enteropathogenic and enterohaemorrhagic diarrhoea (Donnenberg et al., 1993; Schmidt, 2010).

*Listeria monocytogenes*, *C*. *jejuni‐* and STEC can persist in dairy farms for several months, despite good hygienic management. It has been suggested that milk contamination of STEC can be reduced by increased culling rates, improving cleaning and disinfection of barns, and by giving the livestock access to pastures (Castro et al., 2018; Jaakkonen et al., 2019). Poor‐quality silage is believed to be the main reservoir for introducing *L. monocytogenes* to the dairy farm environment (Yoshida et al., 1998). Direct *L. monocytogenes* contamination of raw milk from cows with *Listeria* mastitis may also occur but contamination via the milking instruments, where this pathogen can persist on surfaces, is probably a more relevant route of transmission to raw milk (Borucki et al., 2005; Yoshida et al., 1998). Other studies have shown that *L. monocytogenes* is able to propagate in refrigerated milk during storage (Artursson et al., 2018; Castro et al., 2017, 2018). This is not the case for *Campylobacter* spp. and STEC, but due to low infectious dose, propagation in food matrixes is not necessary for their ability to cause disease in humans (Epps et al., 2013). *Salmonella* spp. were not included in this study as the Norwegian Veterinary Institute performs continuous *Salmonella* surveillance and estimate a prevalence below 0.1% in the Norwegian cattle population. Most (78%–80%) of the human salmonellosis cases in Norway are acquired abroad and are rarely caused by domestically produced food (Norwegian Veterinary Institute, 2019).

Automatic milking systems (AMS) with robotic milking were introduced to European dairy farms in the early 1990s, (Cogato et al., 2021; Jacobs & Siegford, 2012). Since 2000, AMS have become common installations in Norwegian dairy farms and, today, more than 50% of the milk produced in Norway, originates from farms using milking robots (Hansen et al., 2019; Nørstebø et al., 2018). AMS is common in farms with large herds and loose housing where significant contact occurs between animals. This can lead to more problems with faecal contamination and cow cleanliness than experienced in tie‐stall housing systems (Hovinen et al., 2009; Hovinen & Pyörälä, 2011). Other studies have investigated possible connections between farm operational systems and total bacterial count in bulk tank milk (BTM) (de Koning et al., 2003; Klungel et al., 2000; Rasmussen et al., 2001, 2002; van der Vorst & Hogeveen, 2000; Van der Vorst & Ouweltjes, 2003), but to our knowledge; there is limited knowledge on how the transition from tie‐stall to loose housing influence the occurrence of zoonotic bacteria in the farm environment and in BTM. To gain more information on how farm practices and different housing systems influence the safety of raw milk, this study investigated the prevalence of *Campylobacter*, *L. monocytogenes* and STEC in raw milk and environmental samples from dairy farms representing both loose housing and tie‐stall housing systems. The relationship between herd hygienic status and the presence of *L. monocytogenes*, *Campylobacter* spp., and STEC in the farm samples was also evaluated.

## MATERIALS AND METHODS

To assess the risk associated with consuming unpasteurized milk in Norway, aseptic samples of BTM, milk filters and teat milk from Norwegian dairy herds were collected and examined for presence of *L. monocytogenes*, *Campylobacter* ssp. and Shiga toxin‐producing *E. coli* (STEC). Samples were also collected from faeces, feed (forage plants) and teat swabs to examine potential correlations between the presence of pathogens in the raw milk and in the farm environment. A visual evaluation of the hygienic status of the herds was performed by scoring the cleanness of the cattle at each sampling occasion. A total of 18 dairy herds from four different geographical areas, located within 100 km from Oslo, in southeast of Norway were randomly selected from a registry (Brønnøysundregisteret) where all Norwegian dairy‐herds are registered. The milk produced at the farms is used for commercial production of drinking milk, cream, cheese, sour cream, yoghurt and other dairy products. Seven of the herds had tie‐stall housing where the cows are tied up in individual bedding, feeding, and milking stalls. The tie‐stall farms use conventional milking systems with manual application performed by an operator, usually the farmer, at specific times of the day. Eleven of the herds had loose housing where cows share a pen with common bedding, feeding and grooming area. In nine of the loose housed herds, the cows had access to an AMS which they enter voluntarily at any time of the day. One farm had loose housing with an integrated milking parlour operated by the farmer, and there was also a loose housing farm with milking performed on a carousel operated by the farmer. All herds have individual teat washing before milking, and some farms uses post milking teat dipping/spraying to secure udder hygiene. The milk is cooled (4°C) and stored in an on‐farm bulk tank before transported to the dairy within 2–3 days. In farms with loose housing systems, the number of animals ranged from 25 to 120 (mean 63) and in tie‐stall farms from 19 to 33 animals (mean 25). The loose housed cows had access to an outdoor pasture for a minimum of 8 weeks during the sampling period, and the tie‐stalled cows a minimum of 16 weeks. To account for seasonal variations in pathogen occurrence, each farm was sampled six times over a period of 11 months, with some exceptions due to Covid19 restrictions and other technical issues, resulting in variation in total number of samples from the farms. The first sampling was performed in August and September 2019 (one farm in November), the second in November and December 2019, the third in January 2020, the fourth in February–March 2020, the fifth in May 2020 and the sixth in June 2020. Samples from BTM, milk filter, faeces, feed, and teats were collected at each visit, and teat milk samples were added from visit number three. After collection, all samples were kept in closed sample containers to minimize drying and exposure to air, and they were immediately placed in a cooling bag (32 l, 50 × 33 × 41 cm) containing three to four freeze elements. The microbiological analyses were initiated within 6 h after sample collection.

### Collection of samples

#### BTM

A total of 200–400 ml of BTM was collected in sterile 50 ml tubes or in autoclaved 500 ml glass bottles at each farm visit. Fifteen of the farms had a tap connected to the cooling tank where milk could be drained directly into the sample container. Three farms had cooling tanks with an opening on the top, where an autoclaved ladle was used to transfer milk to the sample container.

#### Milk filters

A disposable milk filter sock with a pore size of 100–250 μM is placed between the milking system and the bulk tank. The milk filter socks were replaced every 12–24 h and were collected at each visit. The filters were immediately cut longitudinally into three pieces (1/3 for each analysis) by a sterile scissor and directly placed in three autoclaved glass bottles, containing 200 ml of media specific for enriching either *Listeria*, *E. coli* or *Campylobacter* spp.

#### Faeces

Fresh faecal samples were collected from the floor at 5–10 different places in each animal house and pooled into a sterile stomacher bag to a total amount of minimum 100 g. Clean disposable plastic gloves were used during collection, and the samples were kept cool until analysis.

#### Feed

During each farm visit, approximately 100 g of feed (silage or silage mixture) was collected from 5 to 10 different locations of the feed alley and pooled into a sterile stomacher bag. Clean disposable plastic gloves were used when handling the feed samples.

#### Teat swabs and teat milk

Generally, 10% of the animals in each herd were sampled during each visit. However, at farms holding <50 animals or more than 100, the numbers of sampled animals were limited to 5 and 10 animals, respectively. Autoclaved cotton swabs moistened in peptone water were rubbed several times across all four teats. A new swab was used for each individual animal. Swabs from different animals were then placed into the same Falcon tube containing 15 ml peptone water and the pooled swab samples were considered to represent one herd. The teat milk samples were collected from each quarter, into sterile Falcon tubes by hand milking from the swabbed cows after disinfecting the teats with 70% ethanol. Samples from individual cows were pooled into one sample representing the herd.

### Hygiene scoring of dairy cows

A cleanliness scoring was performed on a minimum of 30% of the dairy cows in each herd. Three distinct zones of the cow; the udder, lower portions of the hind limbs and upper portions of the hind limbs/flanks, were assessed according to a point scale 0–3, where score 0 was clean with little or no evidence of manure, 1 was clean with only slight manure splashing, 2 was dirty, distinct demarcated plaques of manure and 3 was filthy, confluent plaques of manure (Cook, 2002). Further, the score from the three zones were added together to a total score between zero and nine for each cow, and a mean score was calculated for the herd at each visit. A lower score indicates better hygiene.

### Isolation of *L. monocytogenes*

The samples (25 g BTM, 1/3 milk filter, 10 g faeces, 10 g feed, 5 ml teat swab solution and 5 ml teat milk) were cultured for *L. monocytogenes* according to the method published by the Nordic Committee on Food Analysis (NMKL) No 136, 5th ed. 2010. All samples underwent a two‐step, 1:10 enrichment procedure including a primary enrichment in reduced selectivity Half Fraser broth (Oxoid) at 30°C for 24 h, followed by enrichment in full selectivity Fraser broth (Oxoid) at 37°C for 48 h. Cultures from the Fraser enrichments were plated on ‘Agar Listeria according to Agosti and Ottaviani’ (ALOA) and incubated at 37°C for 24–48 h. The concentration of *L. monocytogenes* in BTM and teat milk was assessed by plating 100 μl of the samples directly on ALOA. The plates were incubated at 37°C for 24–48 h before enumeration. Presumptive *L. monocytogenes* colonies from ALOA plates were confirmed after identification of beta‐haemolytic, catalase positive and rhamnose positive, Gram‐positive rods.

### Isolation of thermophilic *Campylobacter* spp.

Qualitative determination of thermotolerant *Campylobacter* was performed according to NMKL No. 119, 3. Ed., 2007, with some modifications. Samples of BTM milk (25 g), milk filters (1/3), faeces (10 g), teat swab solutions (5 ml) and teat milk samples (5 ml) were transferred into Bolton broth (Oxoid) for enrichment in a 1:10 ratio and then incubated at 37°C for 48 h in a 5% CO_2_ atmosphere. The samples were further plated on selective agar mCCDA (modified charcoal cefoperazone deoxycholate agar; Oxoid) and incubated at 42°C for 48 h in a 5% CO_2_ atmosphere. For enumeration, 100 μl of BTM and teat milk were plated on mCCDA and incubated for 48 h at 37°C. Presumptive *Campylobacter* colonies were confirmed as *Campylobacter* spp. when they were catalase and oxidase positive and appeared as motile s‐shaped rods under phase‐contrast microscopy.

### Identification of STEC in samples

For enrichment of *E. coli* from either 25 ml BTM (100 samples), 1/3 of a milk filter (100 samples), or 10 g of faeces (98 samples), the samples were added to 225, 200 or 90 ml, respectively, of modified Tryptone Soya Broth (mTSB) (Oxoid), supplemented with novobiocin (16 μg/ml) according to ISO/TS 13136:2012, and incubated at 37°C for 24 h. Each pre‐culture was then divided into two parts: one part containing 1 ml that was pelleted at 12,000 *g* for 1 min for DNA isolation and polymerase chain reaction (PCR) analysis, and 1 ml for storage at −80°C until use. DNA was purified using DNeasy® Blood and Tissue kit (Qiagen), following the protocol for ‘Purification of Total DNA from Animal Tissues (Spin‐Column Protocol)’. Each DNA sample was examined for the presence of *stx1*, *stx2* and *eae* by PCR as described below. One μl of mTSB‐enrichment cultures from samples positive for either *stx1*, *stx2* or *eae* were spread on CHROMagar STEC plates (CHROMagar Microbiology) by using an inoculation loop of 1 μl and incubated at 37°C for 24 h. CHROMagar STEC differentiate between STEC (mauve/pink colonies) and other Enterobacteriacae (blue colonies) and inhibits growth of Gram‐positive bacteria. Three mauve/pink colonies from each CHROMagar STEC plate were transferred to Sorbitol MacConkey Agar (SMAC) (Oxoid) plates for two purposes; to achieve single colonies for further testing by PCR and for direct identification of STEC of serotype O157 which grow with beige colonies on SMAC. Resulting single colonies isolated from the three SMAC plates were tested by PCR for detection of *stx1*, *stx2* and *eae* to identify putative potentially human pathogenic STEC isolates.

### PCR

The 298 DNA samples (collected as described above) were screened for the presence of *stx1* and *stx2* by PCR by testing 1 μl of the DNA solution isolated from the mTSB sample, using Thermo Scientific DreamTaq PCR Master Mix and 0.2 μM of the corresponding primers (Table S1). The amplification reactions were run separately for *stx1* and *stx2*, and were performed in an Eppendorf Mastercycler using the following program: 3 min initial denaturation at 94°C followed by 30 cycles of denaturation at 94°C for 10 s, annealing at 52°C for 30 s and primer extension at 72°C for 60 s. The amplifications were terminated after a final elongation step of 7 min at 72°C. DNA isolated from *E. coli* O157:H7 strain EDL933 was used as a positive control and autoclaved water as negative control. The PCR fragments were visualized by agarose gel electrophoresis.

Single colonies from the pure cultures on SMAC (transferred from CHROMagar STEC) were dissolved in 100 μl of autoclaved H_2_O, heated for 99°C for 10 min, and 1 μl of this sample was examined for the presence of *stx1*, *stx2* and *eae* using the primers listed in Table S1. The EaeR primer were designed to detect the Alpha, Beta, Gamma, Zeta, Theta and the Delta versions of *eae*. Attempts to determine the serotype of the STECs by PCR were performed using the primers and conditions described by Sánchez et al. (2015). The *E. coli* isolates used as positive controls were of serotype O103 and O157, and autoclaved water was used as negative control.

### Statistical analyses

A database was established in a Microsoft Excel® spreadsheet. After calculating and reviewing data in Excel, using filter functions and pivot analyses, data were transferred to STATA SE/15 (for Windows, StataCorp) for further analyses. Inspection of the variables was performed in STATA using tabulations, calculations of means, medians, standard errors and 95% confidence intervals. The presence of *Campylobacter*, STEC or *L. monocytogenes* in samples were outcome variables in univariable logistic regression analyses and the repeated sampling was generally taken into account by including the herd as a random variable in the regression models. Seasonal variation in the occurrence of pathogens in samples were taken account of by including visits as a fixed variable in the regression models. The effect of hygiene scores on the occurrence of pathogenic bacteria in the different samples was analysed by including visit as a random variable to account for repeated observations. Odds ratios (OR) are given to describe the effect of the binary variables (e.g. tie‐stall versus loose housing) and β‐coefficients are given for continuous predictors (e.g. herd size). A two‐sided Fisher's exact test was used to look for associations between the presence of pathogens in milk filter and in environmental samples (faeces and fodder). Statistical significance was defined as *p* < 0.05.

## Results

### Prevalence of *L. monocytogenes*, *Campylobacter* spp. and STEC on Norwegian dairy farms

*Listeria monocytogenes* was isolated from 79 of 556 samples (14%) and the distribution of positive samples is shown in Table 1. None of the BTM or teat milk samples were positive for *L. monocytogenes,* but it was found in 13% of the milk filters. One farm had four *L. monocytogenes* positive milk filters and it was the only farm that had *L. monocytogenes* positive milk filters during more than one sampling occasion.

**TABLE 1:** Prevalence of *Listeria monocytogenes* in dairy farm samples

Sampling	BTM	MF	Faeces	Silage	Teat swab	Teat milk	Total
Aug./Sept.	0/18 (0)	0/17 (0)	5/16 (31)	4/18 (22)	0/17 (0)		9/86 (10)
Nov./Dec.	0/18 (0)	4/16 (25)	6/18 (33)	6/18 (33)	1/18 (6)		17/88 (19)
Jan	0/18 (0)	2/17 (12)	4/18 (22)	10/18 (56)	1/18 (6)	0/18 (0)	17/107 (16)
Feb./Mar.	0/18 (0)	1/13 (8)	7/18 (39)	6/18 (33)	1/18 (6)	0/18 (0)	15/103 (15)
May	0/14 (0)	2/14 (14)	2/14 (14)	3/14 (21)	2/14 (14)	0/14 (0)	9/84 (11)
June	0/16 (0)	3/16 (19)	6/15 (40)	3/15 (20)	0/13 (0)	0/13 (0)	12/88 (14)
Total	0/102 (0)	12/93 (13)	30/99 (30)	32/101 (32)	5/98 (5)	0/63 (0)	79/556 (14)

*Note:* Prevalence of bulk tank milk‐ (BTM), milk filter‐ (MF), faeces‐, silage‐, teat swab‐ and teat milk‐samples positive for *Listeria monocytogenes*. The numbers given are positive samples/total samples (%). The samples were collected at six different time points between August 2019 and July 2020.

Silage or silage mixture samples collected in January revealed a higher occurrence of *L. monocytogenes* than those collected in August–September (β = 1.48, *p* = 0.03) and June (β = 1.60, *p* = 0.03). The other sample types showed no seasonal differences (Table S2).

The prevalence of *Campylobacter* spp. was 20% among a total of 455 tested samples (Table 2). *Campylobacter* spp. were not detected by direct plating of BTM and teat milk on mCCDA agar. However, *Campylobacter* spp. were detected in 3% of the BTM samples and 3% of the teat milk samples and in 4% of the milk filter samples after enrichment in Bolton broth. Among faecal samples, 68% were positive for *Campylobacter* spp. All farms had at least one *Campylobacter* spp. positive faecal sample during the sampling period and four farms were positive during all sampling occasions. There was no seasonal variation in the total number of samples containing *Campylobacter* spp. but the periodic sampling revealed a higher detection rate of *Campylobacter* spp. in faeces during visit two/November–December and during visit five/May (Table 2, Table S3).

**TABLE 2:** Prevalence of *Campylobacter* spp. in dairy farm samples

Sampling	BTM	MF	Faeces	Teat swab	Teat milk	Total
Aug./Sept.	2/18 (11)	2/17 (12)	9/16 (56)	1/17 (6)		14/68 (21)
Nov./Dec.	1/18 (6)	1/16 (6)	15/18 (83)	2/18 (11)		19/70 (27)
Jan	0/18 (0)	0/17 (0)	11/18 (61)	3/18 (17)	1/18 (6)	15/89 (17)
Feb./Mar.	0/18 (0)	1/13 (8)	12/18 (67)	3/18 (17)	1/18 (6)	17/85 (20)
May	0/14 (0)	0/14 (0)	12/14 (86)	3/14 (21)	0/14 (0)	15/70 (21)
June	0/16 (0)	0/16 (0)	8/15 (53)	1/13 (8)	0/13 (0)	9/73 (12)
Total	3/102 (3)	4/93 (4)	67/99 (68)	13/98 (13)	2/63 (3)	89/455 (20)

*Note:* Prevalence of bulk tank milk‐ (BTM), milk filter‐ (MF), faeces, teat swab‐ and teat milk‐samples positive for *Campylobacter* spp. The numbers given are positive samples/total samples (%). The samples were collected at six different time points between August 2019 and July 2020.

The frequency of BTM‐samples, milk filter‐samples and faecal‐samples that were PCR positive for *stx1*and/or *stx2* and/or *eae* are given in Table 3. The highest proportion of *stx* positive samples was found in faeces where 34 out of 98 samples (35%) were positive for either *stx1*, *stx2* or both. Among 100 milk filters and 100 BTM‐samples, 27% and 16% respectively, were positive for either *stx1*, *stx2* or for both. In total, 12% of the milk filter samples and 10% of all samples were positive for both *stx* and *eae*.

**TABLE 3:** Detection of *stx1*, *stx2* and *eae* in dairy farm samples

Sample	Visit	*stx1*	*stx2*	*eae*	*stx1/2* + *eae*
Faeces	Aug./Sept.	5/15 (33)	9/15 (60)	3/15 (20)	3/15 (20)
	Nov./Dec.	6/18 (33)	8/18 (44)	4/18 (22)	4/18 (22)
	Jan.	3/18 (17)	5/18 (28)	3/18 (17)	3/18 (17)
	Feb./Mar.	1/18 (6)	5/18 (28)	2/18 (11)	1/18 (6)
	May	2/14 (14)	3/14 (21)	1/14 (7)	1/14 (7)
	June	2/15 (13)	2/15 (13)	1/15 (7)	1/15 (7)
	Total	19/98 (19)	32/98 (33)	14/98 (14)	13/98 (13)
Milk filter	Aug./Sept.	2/18 (11)	7/18 (39)	5/18 (28)	3/18 (17)
	Nov./Dec	2/18 (11)	4/18 (22)	5/18 (28)	2/18 (11)
	Jan.	1/18 (6)	3/18 (17)	4/18 (22)	2/18 (11)
	Feb./Mar.	2/16 (13)	2/16 (13)	3/16 (19)	2/16 (13)
	May	1/14 (7)	3/14 (21)	0/14 (0)	0/14 (0)
	June	1/16 (6)	6/16 (38)	7/16 (44)	3/16 (19)
	Total	9/100 (9)	25/100 (25)	24/100 (24)	12/100 (12)
Bulk tank milk	Aug./Sept.	0/18 (0)	2/18 (11)	5/18 (28)	0/18 (0)
	Nov./Dec.	0/18 (0)	1/18 (6)	2/18 (11)	0/18 (0)
	Jan.	0/18 (0)	0/18 (0)	2/18 (11)	0/18 (0)
	Feb./Mar.	1/18 (6)	2/18 (11)	1/18 (6)	1/18 (6)
	May	0/14 (0)	1/14 (7)	1/14 (7)	0/14 (0)
	June	9/14 (64)	4/14 (29)	4/14 (29)	3/14 (21)
	Total	10/100 (10)	10/100 (10)	15/100 (15)	4/100 (4)
All samples	Total	38/298 (13)	67/298 (22)	53/298 (18)	29/298 (10)

*Note:* Prevalence of bulk tank milk‐samples, milk filter‐samples and faecal‐samples positive for *stx1*, *stx2* and *eae* after enrichment in mTSB at 37°C for 24 h. The numbers given are positive samples/total samples (%).

Sixty‐five out of 99 samples that were PCR positive for either *stx1*, *stx2* and/or *eae* presented typical mauve colonies on Chromagar STEC plates. Subsequent PCR analysis of single colony isolates revealed that 19 of 65 isolates were positive for either *stx1 or stx2,* or a combination of *stx1* and *stx2* and were, therefore, regarded as STECs (Table 4). None of the 19 *stx* positive isolates presented beige colonies on SMAC plates, indicating other serotypes than O157:H7. Out of 298 tested samples, STEC were isolated from 6% (19) of the samples. Muliplex PCR, targeting 21 of the most clinically relevant serogroups for STEC infections in humans, revealed that the STECs isolated in this study did not belong to any of the seven most common serotypes O26, O45, O103, O111, O121, O145, O157, nor to the 14 remaining tested serotypes (Table S1).

**TABLE 4:** Isolation of Shiga toxin‐producing *Escherichia coli* from BTM, milk filters and faeces

Sampling	BTM	MF	Faeces	Total all samples
Aug./Sept.	0/18 (0)	1/18 (6)	1/15 (7)	2/51 (4)
Nov./Dec.	0/18 (0)	1/18 (6)	3^a^/18 (17)	4/54 (7)
Jan	0/18 (0)	1/18 (6)	2^a^/18 (11)	3/54 (6)
Feb./Mar.	0/18 (0)	0/16 (0)	1/18 (6)	1/52 (2)
May	0/14 (0)	1/14 (7)	2/14 (14)	3/42 (7)
June	1/14 (7)	3^a^/16 (19)	2^a^/15 (13)	6/45 (13)
Total	1/100 (1)	7/100 (7)	11/98 (11)	19/298 (6)

*Note:* Prevalence of Shiga toxin producing *Escherichia coli* isolates from bulk tank milk (BTM) samples, milk filter (MF) samples and faecal samples positive for *stx1*, *stx2* and *eae*. The numbers given are positive samples/total samples (%).

^a^ *Escherichia coli* isolates positive for both *stx* and *eae* were isolated from three faecal samples (sampling 2, 3 and 6) and from one milk filter (sampling 6).

Four out of 19 STEC isolates (21%) were positive for *eae* and were therefore considered as high‐risk isolates. Three of these isolates were from the same farm and collected from two faecal samples and one milk filter sample. The fourth isolate was isolated from a faecal sample from another farm. Both farms were using loose housing.

A higher prevalence of *stx2* positive faeces‐samples was observed in the autumn compared with the spring and early summer months (Table 3). The differences between visit one (August–September) and visit five (May) (β = −1.70, *p* = 0.01) and six (June) (β = −2.28, *p* = 0.02) were statistically significant (Table S4). The highest seasonal variation in the prevalence of STEC during the sampling period was observed in milk filters between visit one (August–September) and visit five (May) (β = −0.27, *p* = 0.053) (Table S5). The prevalence of *eae* positive BTM samples was higher during visit one in August–September and visit six in June compared with the other samplings (Table 3) with a statistically significant difference between visit six and visit four (β = 1.92, *p* = 0.05) (Table S4). This was not the case for *eae* in the faecal samples, where the highest level of positive samples was observed in August to December (Table 3). However, the *eae* levels were relatively high in both faeces and BTM at visit one (August/September) (Table 3).

To summarize the results, *Campylobacter* spp. were at some point isolated from all farms and all these farms, except farm 18, had one or more positive *L. monocytogenes* samples, and six farms had one or more samples positive for STEC. A summary of these findings is shown in Table S6.

### The prevalence of pathogens in samples from loose housing herds compared with tie‐stall herds

**FIGURE 1:**
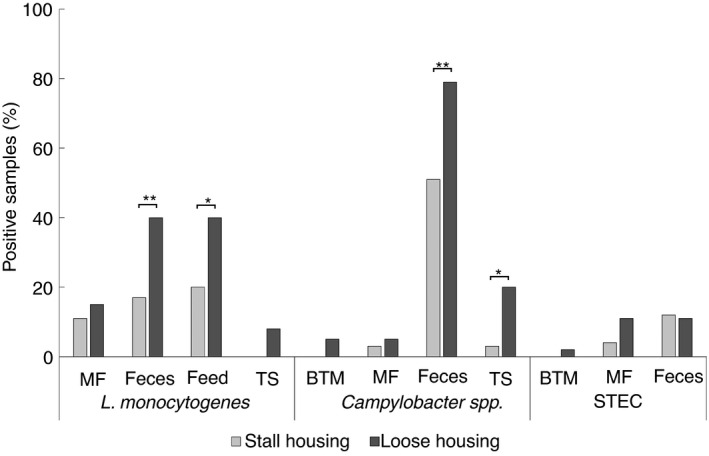
Pathogen occurrence according to housing system. Number of (%) samples positive for *Listeria monocytogenes*, *Campylobacter* spp. and Shiga toxin‐producing *Escherichia coli* in loose housing versus tie‐stall housing (*; *p* < 0.05, **; *p* < 0.02). MF, milk filter; BTM, bulk tank milk; TS, teat swab

*L. monocytogenes* was detected more frequently in faecal samples from loose housing herds compared with tie‐stall herds (OR = 3.19, *p* = 0.02) (Table S2), with an isolation prevalence of 40% and 15% respectively (Figure 1). *L. monocytogenes* was isolated more frequently from feed samples in farms with loose housing systems compared with tie‐stall farms (OR = 2.75, *p* = 0.03) (Figure 1).

*L. monocytogenes* was isolated from milk filters from nine out of 18 farms and there was no difference in occurrence between farms with loose stall housing systems compared with tie‐stall housing systems (Figure 1). Notable, the herd which had *L. monocytogenes* positive milk filters during four sampling occasions had loose housing system. Milk filters were significantly more often positive for *L. monocytogenes* when a faecal sample (OR = 6.6, *p* < 0.01) or feed sample (OR = 8.9, *p* < 0.01) was positive for *L. monocytogenes* at the same sampling occasion. A positive association between herd size and the presence of *L. monocytogenes* in faecal samples was observed (*p* < 0.01).

There was a significant difference in the occurrence of *Campylobacter* spp. in faecal samples from farms with loose housing systems compared with tie‐stall housing (OR = 3.65, *p* < 0. 01) (Figure 1; Table S3). Similarly, there was a higher occurrence of *Campylobacter* spp. in teat swabs from farms with loose housing compared with tie‐stall housing farms (OR = 9.70, *p* = 0.03). There was, however, no significant difference in the prevalence of *Campylobacter* spp. in milk filters (*p* = 0.52) or teat milk samples (*p* = 0.76) between farms having loose housing versus tie‐stall systems. Neither farms with loose housing nor those with tie‐stall housing showed an association between herd size and the occurrence of *Campylobacter* species in faeces samples. There was, however, an association between the isolation rate of *Campylobacter* spp. in teat swabs (β = 0.03, *p* < 0.01) and herd size regardless of housing system.

*Campylobacter* spp. was isolated from milk filters from four out of 18 herds; one of these had tie‐stall housing and three had loose housing. A two‐sided Fisher exact test did not show an association between positive faecal samples and positive milk filter samples (OR = 1.02, *p* = 1.00), but there were too few positive milk filters to look for a correlation with environmental samples.

Seven of the 19 STECs were isolated from tie‐stall herds and 12 of the isolates were from loose housing herds. STECs were isolated from faecal samples collected from four loose housing herds and from two tie‐stall herds. However, four out of 11 STEC positive faecal samples (36%) came from one specific farm where the animals were tie stalled. STECs were also isolated from seven milk filters distributed over 5 out of 18 herds; one of these herds had tie‐stall housing while four had loose housing. Notably, one STEC positive BTM sample was collected from a loose housed herd. The four *stx* and *eae* positive samples were collected from loose housing herds.

### Association between dairy cow hygiene score and detection of pathogenic bacteria in dairy farm samples

**FIGURE 2:**
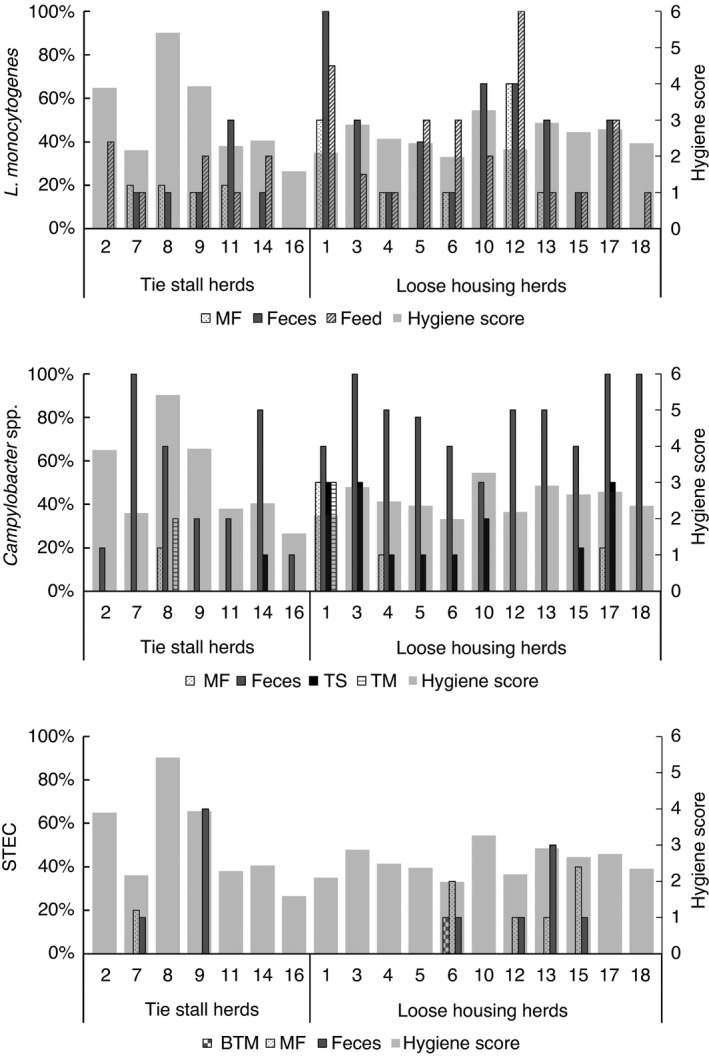
Pathogen occurrence versus dairy cattle hygiene score. % samples positive for *Listeria monocytogenes*, *Campylobacter* spp. and Shiga toxin‐producing *Escherichia coli* in each herd together with average dairy cattle hygiene score. Score points (0–3) from three body zones were summarized, giving a hygiene score between zero and nine for each cow. The average dairy cattle hygiene score was calculated as the average of hygiene scores within one herd from four to six visits. No herds had an average dairy cattle hygiene score higher than six. MF = milk filter, BTM = bulk tank milk, TS = teat swab, TM = teat milk

During sampling, the hygienic status of each cattle herd was scored (0–9) and the mean score from four to six sampling occasions are shown in Figure 2. We observed an association (β = 0.83, *p* = 0.03) between dairy cow hygiene score and detection of *Campylobacter* spp. in teat milk samples (Figure 2; Table S3). No association between hygiene score and detection of *L. monocytogenes* or *Campylobacter* species in BTM, milk filter, faeces, feed or teat swab was observed (Figure 2). Furthermore, no correlation was seen between dairy cow hygiene score and detection of STEC from BTM, milk filters or faeces. Interestingly, the farm with the lowest dairy cow hygiene score had the third lowest *L. monocytogenes* detection rate (6% positive), and STEC was not detected in any of the samples from this farm. *Campylobacter* spp. were detected in 24% of the samples from this herd, the fifth highest detection rate of all farms included in the study.

## DISCUSSION

To explore the potential risk associated with consumption of UPM in Norway, the occurrence of *L. monocytogenes*, *Campylobacter* ssp. and STEC in Norwegian dairy herds and in raw milk was examined. Eighteen different farms, located in a radius of 100 km around Oslo, were included in the study. The included farms are regarded representative for this region but may not represent the total dairy cattle population in Norway due to geographical and climatic differences. To generalize upon the entire Norwegian population, future studies should include additional farms from different parts of Norway.

Consumption of milk and dairy products has been associated with approximately half of all foodborne *L. monocytogenes* outbreaks in Europe, which makes it a serious public health concern (De Buyser et al., 2001; Lundén et al., 2004). In this study, *L. monocytogenes* was isolated from 13% of the milk filters but it was not found in any of the BTM samples. A similar occurrence was reported from a Swedish study from 2018 which detected *L. monocytogenes* in 7% of the milk filter samples but not in the BTM samples (Artursson et al., 2018). Studies from other European countries have found *L. monocytogenes* in UPM samples with a prevalence of 1%–4% (Beckers et al., 1987; Waak et al., 2002). A higher prevalence of *L. monocytogenes* was reported from a Finnish study, which found *L. monocytogenes* in 29% of milk filter samples and 13% of BTM samples from three dairy farms (Castro et al., 2018). An American study from 2018, found *L. monocytogenes* in 2.5% of milk filter samples and in 1.1% BTM samples (Sonnier et al., 2018), which is similar to what was reported from European studies (Artursson et al., 2018; Beckers et al., 1987; Waak et al., 2002). The detection of *L. monocytogenes* in the milk filter samples in all these studies strongly indicate that this bacterium can be present in milking systems. The low prevalence of *L. monocytogenes* detected in BTM in the present study is most likely due to a dilution effect and small testing volumes and do not exclude the presence of *L. monocytogenes* in BTM. The absence of *L. monocytogenes* in teat milk is in accordance with *Listeria* being an environmental contaminant introduced to farm buildings through silage harvest or faecal shredding rather than being a component of the normal udder flora, which supports the importance of good milking hygiene.

In this study, we detected *Campylobacter* spp. in 4% of the milk filter samples, in 3% of the BTM samples, and in 68% of the faecal samples. For comparison, a study from Finland reported the prevalence of *C. jejuni* in milk filter samples to be less than 1%. In the Finnish study, it was not found in BTM samples but was present in 53% of faecal samples (Jaakkonen et al., 2019). In a Swedish study, *C. jejuni* was detected in 7% of milk filters but not in BTM samples (Artursson et al., 2018). The farms included in the Finnish study (Jaakkonen et al., 2019) tested positive for *C. jejuni* and STEC O157:H7 prior to the study took place and had already introduced strict hygienic measures to get rid of the problem, which might have led to underestimation of the pathogen‐prevalence relative to more normal settings. In the Finnish and the Swedish study, the identity of *C. jejuni* was confirmed by MALDI biotyping and pulsed‐field gel electrophoresis (PFGE), respectively, but in the present study, it was only identified to the level of ‘thermophilic *Campylobacter* spp.’ which may also include other *Campylobacter* spp. than *C. jejuni*.

Campylobacteriosis has for many years been the most commonly reported gastrointestinal disease in the EU (European Food Safety Authority and European Centre for Disease Prevention and Control, 2018), and outbreaks associated with consumption of UPM have frequently been reported (Harrington et al., 2002; Heuvelink et al., 2009; Kenyon et al., 2020; Lehner et al., 2000; Schildt et al., 2006). In 2017, 66 Danish school children got campylobacteriosis after visiting a farm where they had raw milk served directly from the barn (Statens Serum Institut, 2018). A similar outbreak occurred in Sweden in 2014, where 11 people, seven of them young children, fell ill after consumption of UPM after visiting a dairy farm (Lahti et al., 2017). Altogether, based on the current and previous studies there is a risk of contracting campylobacteriosis after consumption of UPM.

One of the most important health‐threats associated with consumption of UPM is STEC. Cattle are a natural reservoir of STEC, and approximately 75% of STEC outbreaks are linked to consumption of contaminated beef and milk products (Sperandio & Nguyen, 2012). This study showed an STEC occurrence of 7%, 1% and 11% in milk filter, BTM and faeces samples respectively. We also observed a tendency for a higher prevalence of *stx2* genes and STEC in the faeces samples collected in August–September (visit one) compared with samples collected in May (visit five). The European Union summary report on trends and sources of zoonoses, zoonotic agents and food‐borne outbreaks announce that 8.1% of European cattle tested positive for STEC in 2017 (European Food Safety Authority and European Centre for Disease Prevention and Control, 2018), which is similar to what was found in the present study. In the before‐mentioned Finnish study, Jaakkonen et al. (2019) isolated 2% and 0% of STEC O157 and 1% and 0% of non‐O157 STECs from milk filters and BTM, respectively, which is a slightly lower occurrence than observed in the present study. We have, however, used a different approach to identify STEC than was used in the Finnish study, as we omitted the immunomagnetic separation step, which selects for certain serotypes. The inclusion of all *stx* positive isolates, regardless of serotype, could at least partly explain the higher STEC prevalence obtained in this study. The first described *E. coli* causing enterohaemorrhagic disease and HUS was of serotype O157:H7 (Riley et al., 1983) but non‐O157 STEC infections have increasingly been reported over the last decade (Brooks et al., 2005; Gould et al., 2013; Hughes et al., 2006). Since new STEC variants are continuously emerging, all serotypes should be considered as potential pathogens (Bielaszewska et al., 2011; Rasko et al., 2011). Notably, even the presence of low levels of STEC in UPM can pose a serious risk, particularly for individuals belonging to the high‐risk group as it has a low infectious dose of only 10–100 bacteria (Sperandio & Nguyen, 2012).

The primer‐panel used for geno‐serotyping was described by Sánchez et al. (2015), and was designed to identify 21 clinically relevant serogroups of STEC. It was, however, not possible to identify the serotypes of the STECs isolated in this study by using this primer panel, which indicate that they belong to other serotypes than those that are identified by this method. Notably, as many as 187 *E. coli* serogroups have been described based on nucleotide sequences of the O‐antigen gene cluster (DebRoy et al., 2016) and, out of these, 158 are known to carry the Shiga toxin gene(s) (Ludwig et al., 2020).

Previous studies have reported *stx* gene prevalences of 7%–15% for BTM samples and 40%–50% for milk filter samples (Jaakkonen et al., 2019; Van Kessel et al., 2011). In the present study, 20% of all BTM samples and 34% of all milk filter samples were PCR positive for *stx*. Notably, as *stx* genes are carried by bacteriophages, free phage particles will also result in a positive detection when PCR screening samples. Therefore, it is important to keep in mind that food samples that are PCR positive for *stx*, do not necessarily represent a direct risk to human health but should rather be interpreted as a sign of increased risk of occurrence of STEC. Intimin, encoded by *eae*, is necessary for intimate attachment of enteropathogenic *E. coli* (EPEC) to epithelial cells (Donnenberg et al., 1993). Approximately 25% of the milk filter samples in this study were positive for *eae*, indicating a high likelihood for the presence of Intimin positive *E. coli* isolates (also called enteropathogenic *E. coli*) in the raw milk. This study also identified an *eae* positive STEC isolate from a milk filter sample, indicating a high possibility of presence of STEC in raw milk. The lack of significant association between the *eae* content in faeces and in BTM observed during the year may be due to the size of the study, and larger studies are needed to address if detection of *eae* in BTM coincides with a high detection rate of *eae* in faeces. Summer and autumn season have been shown to be significant risk factors for human STEC infections (European Centre for Disease Prevention and Control, 2021; Mughini‐Gras et al., 2018), and cattle have been shown to excrete more in warm temperatures (Venegas‐Vargas et al., 2016). The current study indicates a similar pattern for dairy cattle in Norway, as *stx2* were significantly more prevalent in faeces in the autumn compared to spring and early summer, and *eae* in BTM were significantly more prevalent in summer and early autumn compared to the other samplings. Although the findings of this study indicate a higher prevalence of STEC shedding during summer and autumn season further studies are needed to conclude.

To explore the differences in pathogen occurrence in farms with different operating systems both tie‐stall and loose stall herds were included in the study. Statistical analysis revealed that the occurrence of *Campylobacter* spp. in faeces and teat swabs and *L. monocytogenes* in faeces and feed was higher in loose housed herds compared with tie‐stall herds. Confounding factors, like herd size, may at least partly explain the difference in occurrence as loose housed herds often are of larger size compared with tie‐stalled, which confers more animal‐to‐animal interactions and increased faecal contamination of the environment.

The hygiene of dairy cows can be used as an indicator of animal welfare and the quality of the farm facilities (Hultgren & Bergsten, 2001; Welfare Quality Consortium, 2009) and poor hygiene are associated with an increased occurrence of mastitis and high somatic cell counts (Cook & Reinemann, 2006; Schreiner & Ruegg, 2003). Poor udder hygiene has been associated with dirty environment (Devries et al., 2012) and pathogens are shown to be transmitted to milk via dirt from the udder (Vissers et al., 2007). Our study indicates an association between cow hygiene and detection of *Campylobacter* spp. in teat milk samples. The cow hygiene is likely to depend on the state of the surrounding environment during the different seasons. An Italian study reports that cows were significantly dirtier in December, January and February compared with April and October and they suggested that difficulties in keeping the bedding dry during the rainy season resulted in an increased amount of manure on legs, flanks, and udders (Zucali et al., 2011).

The feed samples showed a seasonal variation in the presence of *L. monocytogenes,* with higher levels in the winter months November/December, January, and February/March (33%, 56% and 33% respectively) compared with August/September, May, and June (22%, 21% and 20% respectively). Notably, only January compared with September and June were statistically significant (*p* = 0.03). Similar seasonal variations were also reported by a Finnish study which detected higher levels of *L. monocytogenes* in milk filters during the indoor season (Castro et al., 2018). A study from New York state (USA), reported a higher prevalence of *L. monocytogenes* during the winter season in samples collected from cattle and small‐ruminant farms (Nightingale et al., 2005). However, there are also reports which did not find any seasonal variations in the prevalence of *L. monocytogenes* at dairy farms (Gaya et al., 1998; Hassan et al., 2001) and some studies found higher *L. monocytogenes* levels during the summer season (Dalzini et al., 2016; Hutchison et al., 2005). Differences in study design and local climate conditions could be factors that account for the discrepancy regarding seasonal variations in *L. monocytogenes* levels reported from different studies. Dairy cattle grazing practices in Norway varies across climatic zones, and the farms included in this study were located in a typical inland climate, characterized by a relatively short grazing season. In this region, silage is provided both during housing‐ and grazing seasons in combination with concentrates to compensate for feed intake, feed quality and nutritional requirements according to the individual milk production. The silage is generally stored in sealed bales, silos or in silage pits until use. Associations between feeding practices, silage storage methods, feed composition and *L. monocytogenes* contamination were not part of the current investigation.

In conclusion, the present study reveals a wide distribution of *L. monocytogenes*, *Campylobacter* spp. and STEC in environmental samples collected at Norwegian dairy farms, independent of housing system. The presence of bacteria with low infectious doses, such as *Campylobacter* spp. and STEC, in milking systems combined with a human population of increasing age and with more people suffering from underlaying risk factors for severe disease, reinforce the importance of strict regulations regarding commercial sales of UPM. The evolvement of agricultural technologies will most probably continue to present new food safety challenges in the future and the need for continuous adaptation of hygiene measures and pathogen control strategies must be highlighted.

## CONFLICT OF INTEREST

No conflict of interest declared.

## Supporting information


Tables S1–S6

## Data Availability

The datasets supporting the conclusions of this article are included within the article and its additional files.
