# Supplementary material for: The prevalence of Campylobacter spp., Listeria monocytogenes and Shiga toxin‐producing Escherichia coli in Norwegian dairy cattle farms: A comparison between free stall and tie stall housing systems
Source: J Appl Microbiol. 2022 Mar 11;132(5):3959–72. doi: 10.1111/jam.15512 (PMC9315008; doi:10.1111/jam.15512)
Supplement: Supplementary file 1 — Tables S1–S6 [file JAM-132-3959-s001.pdf]

Table S1 Primers used in the study

Primer name	Primer sequence (5´-3´)	Reference
Stx2F	GCGTTTTGACCATCTTCGT	Muniesa et al. 1998
Stx2R	ACAGGAGCAGTTTCAGACAG	Muniesa et al. 1998
Stx1F (stx1M16625f)	GATAGTGGCTCAGGGGATAAT	Sekse et al. 2005
Stx1R	GCCGAAAACGTAAAGCTTCAG	This study
EaeF	GTGGCGAATACTGGCGAGACT	Paton et al. 1998
EaeR	CTTGTGCGCTTTGGCTTC	This study
O5F	CTTATCCGATTAATGGCTTC	Sánchez et al. 2015
O5R	TAGTCGATTTGCTTTTATGG	Sánchez et al. 2015
O91F	TTTTCTGGAATGCTTGATGA	Sánchez et al. 2015
O91R	ATAATTTTACGCCGTGTTTG	Sánchez et al. 2015
O26F	ACTCTTGCTTCGCCTGTT	Monday et al. 2007
O26R	CAGCGATACTTTGAACCTTAT	Monday et al. 2007
O103F	TATCCTTCATAGCCTGTTGTT	Monday et al. 2007
O103R	TTATAATAGTAATAAGCCAGACACC	Sánchez et al. 2015
O145F	TTGAGCACTTATCACAAGAGATT	Monday et al. 2007
O145R	GATTGAATAGCTGAAGTCATACTAAC	Monday et al. 2007
O121F	GTAGCGAAAGGTTAGACTGG	Monday et al. 2007
O121R	ATGGGAAAGCTGATACTGC	Monday et al. 2007
O111F	GTTGCGAGGAATAATTCTTCA	Monday et al. 2007
O111R	CCATAGATATTGCATAAAGGC	Monday et al. 2007
O55F	ATCGCAATTGCAATAAACTC	Sánchez et al. 2015
O55R	CCCAACTCTAGTAGATAAAAGCC	Sánchez et al. 2015
O128F	TTTCGATCGTCTTGTTCAGG	Sánchez et al. 2015
O128R	CAATGGGCAATTAACACAGAG	Sánchez et al. 2015
O113F	TAACGGGATTAGAAGTGGAT	Sánchez et al. 2015
O113R	ATATAAGGCAGAAATGAGAGG	Sánchez et al. 2015
O146F	ATCAGTTCATGGGTTGTATTC	Sánchez et al. 2015
O146R	AGGAACATGGATGAAAGAAG	Sánchez et al. 2015
O45F	GACTTTCGTTGCGTTGTG	Sánchez et al. 2015
O45R	CTGCAAGTGTAGCGAAAAC	Sánchez et al. 2015
O177F	TCGGTGTTTGAAGGGGAAG	Sánchez et al. 2015

O177R	GTCCATGCATATGCCGTTC	Sánchez et al. 2015
O157F	CTCAATTTATAAAAAAGACGCTC	Sánchez et al. 2015
O157R	TCCAAATATTAACGACTTCACTAC	Sánchez et al. 2015
O15F	GCGTTGCCTACTTACTTATTATC	Sánchez et al. 2015
O15R	ATGCAAGTCCAGCCAAAC	Sánchez et al. 2015
O104F	CGGTGTATTAAGAAGTGTTGTC	Sánchez et al. 2015
O104R	ATACTCCCCATAGAAACGC	Sánchez et al. 2015
O118F	TGGAGAACAGATAGCAAGAGG	Sánchez et al. 2015
O118R	TATCCGACAAACACGAACC	Sánchez et al. 2015
O123F	GAAAGAACAGAATCAGACTATGC	Sánchez et al. 2015
O123R	TGTGCTAGCGCTAAAGGAC	Sánchez et al. 2015
O165F	AACTGTTTATCCGAAGTGGTAG	Sánchez et al. 2015
O165R	CACGCTTTAACGCATACAG	Sánchez et al. 2015
O172F	ATTGGGTAGCCTCAGTAAAG	Sánchez et al. 2015
O172R	CAGTCCAAACAGTGACAGTATC	Sánchez et al. 2015

Table S2 Statistics on *L. monocytogenes* occurrence

**Predictor variable**	**Random effect**	**Outcome variable**	**Odds ratio**	**Coefficient**	**Std. Err.**	**z**	**P-value**	**95% Lo**	**95% Hi**
Loose vs tie stall housing		Feces	3.19		1.58	2.35	0.02	1.21	8.41
		Silage	2.75		1.30	2.13	0.03	1.09	6.96
		Milk filter	1.45		0.94	0.57	0.57	0.40	5.20
Herd size	Herd number	Feces		0.03	0.01	4.12	<0.01	0.02	0.05
		Silage		0.01	0.01	1.84	0.07	<-0.01	0.03
		Milk filter		0.01	0.01	0.68	0.50	-0.01	0.03
		Teat swab		0.02	0.01	1.83	0.07	0.00	0.04
Dairy cow cleanliness score 0-9	Visit	Feces		-0.17	0.18	-0.98	0.33	-0.52	0.18
		Milk filter		-0.20	0.28	-0.74	0.46	-0.75	0.34
		Teat swab		-0.11	0.37	-0.30	0.76	-0.83	0.61
Visit 3 vs 1	Herd number	Silage		1.48	0.68	2.18	0.03	0.15	2.80
Visit 3 vs 6				1.6	0.75	2.15	0.03	0.14	3.08
Feces* pos or neg		Milk filter	6.6				<0.01	1.50	32.49
Feed* pos or neg			8.85				<0.01	1.91	54.22

*Fisher exact test

Table S3 Statistics on *Campylobacter* spp. occurrence

**Predictor variable**	**Random effect**	**Outcome variable**	**Odds ratio**	**Coefficient**	**Std. Err.**	**z**	**P-value**	**95% Lo**	**95% Hi**
Loose vs stall housing		Feces	3.65		1.64	2.88	<0.01	1.51	8.82
		Teat milk	0.65		0.93	-0.30	0.76	0.04	10.87
		Milk filter	2.13		2.51	0.65	0.52	0.21	21.34
		Teat swab	9.70		10.32	2.14	0.03	1.21	78.00
Herd size	Herd number	Feces		0.01	0.01	0.74	0.46	-0.02	0.04
		Teat milk		0.02	0.02	0.97	0.33	-0.02	0.07
		Milk filter		0.02	0.01	1.40	0.16	-0.01	0.05
		Bulk tank milk		0.03	0.01	2.24	0.03	<0.01	0.05
		Teat swab		0.03	0.01	4.59	<0.01	0.02	0.04
Dairy cow cleanliness score	Visit	Feces		-0.09	0.17	-0.56	0.58	-0.43	0.24
		Teat milk		0.83	0.38	2.21	0.03	0.10	1.57
		Milk filter		0.56	0.30	1.86	0.06	-0.03	1.15
		Bulk tank milk		-0.02	0.46	-0.05	0.96	-0.93	0.89
		Teat swab		-0.14	0.24	-0.59	0.56	-0.61	0.33
Season	Visit 2 vs 1	Feces		1.36	0.57	2.37	0.018	0.23	2.48
	Visit 2 vs 6			1.48	0.76	1.95	0.051	-0.01	2.96
	Visit 5 vs 1			1.54	0.74	2.08	0.037	0.09	2.99
	Visit 5 vs 6			1.66	0.72	2.29	0.022	0.24	3.08

Table S4 Statistics on *stx* and *eae* occurrence

Predictor variable	Random effect	Outcome variable		Odds ratio	Coefficient	Std. Err.	z	P-value	95% Lo	95% Hi
Loose vs stall housing		Feces	*stx*	1.04		0.45	0.10	0.92	0.45	2.42
			eae	1.97		1.24	1.07	0.28	0.57	6.78
		Milk filter	*stx*	1.85		0.90	1.28	0.20	0.72	4.78
			eae	3.24		1.79	2.13	0.03	1.10	9.59
		Bulk tank milk	*stx*	1.57		0.92	0.78	0.44	0.50	4.93
			eae	5.26		4.15	2.10	0.04	1.12	24.73
Herd size		Feces	*stx*	0.99		0.01	-1.07	0.28	0.98	1.01
			eae	1.00		0.01	0.41	0.68	0.99	1.02
		Milk filter	*stx*	0.995		0.01	-0.56	0.58	0.98	1.01
			eae	1.01		0.01	1.48	0.14	1.00	1.03
		Bulk tank milk	*stx*	0.99		0.01	-1.17	0.24	0.96	1.01
			eae	1.00		0.01	0.08	0.94	0.98	1.02
Dairy cow cleanliness score	Visit	Feces	*stx*		0.12	0.16	0.76	0.45	-0.19	0.43
			eae		0.37	0.20	1.87	0.06	-0.02	0.76
		Milk filter	*stx*		-0.19	0.19	-1.02	0.31	-0.56	0.18
			eae		-0.01	0.19	-0.07	0.95	-0.38	0.35
		Bulk tank milk	*stx*		-0.41	0.35	-1.19	0.24	-0.01	0.27
			eae		-0.28	0.25	-1.09	0.27	-0.77	0.22

Visit 5 vs 1		Feces	*stx2*		-1.70	0.61	-2.77	0.006	-2.91	-0.50
Visit 6 vs 1		Feces	*stx2*		-2.28	0.97	-2.35	0.019	-4.17	-0.38
Visit 4 vs 1		Bulk tank milk	*stx1*		-3.42	1.10	-3.12	0.002	-5.57	-1.27
Visit 6 vs 4		Bulk tank milk	*eae*		1.92	0.96	1.99	0.047	0.03	3.81

Table S5 Statistics on STEC occurrence

Predictor variable	Random effect	Outcome variable	Odds ratio	Coefficient	Std. Err.	z	P-value	95% Lo	95% Hi
Loose vs stall housing		Milk filter	4.33		4.77	1.33	0.18	0.50	37.45
		Feces	0.85		0.55	-0.26	0.80	0.24	2.99
Herd size	Herd number	Milk filter		-0.02	0.02	-1.29	0.20	-0.05	0.01
		Feces		-0.01	0.02	-0.82	0.41	-0.05	0.02
Dairy cow cleanliness score 0-9	Visit	Milk filter		-0.42	0.37	-1.15	0.25	-1.15	0.30
		Feces		0.21	0.21	1.00	0.32	-0.20	0.63
Visit 5 vs 1	Herd number	Milk filter		0.27	0.14	1.94	0.053	<-0.01	0.54

Table S6 Number positive samples from the individual dairy farms

Farm	*L. monocytogenes*	*Campylobacter* spp.	STEC
1*	8/19	7/15	0/11
2	2/26	1/21	0/14
3*	4/20	6/16	0/12
4*	3/34	8/28	0/17
5*	5/33	5/27	0/17
6*	5/34	5/28	4/18
7	3/33	6/27	2/17
8	2/31	6/25	0/18
9	4/34	2/28	4/18
10*	6/33	5/27	0/18
11	5/33	2/27	0/18
12*	15/34	5/28	2/18
13*	6/34	5/28	4/18
14	3/34	6/28	0/18
15*	3/32	5/26	3/17
16	0/34	1/28	0/18
17*	4/24	8/20	0/13
18*	1/34	6/28	0/18
Total	79/556	89/455	19/298

Number of dairy farm samples positive for *L. monocytogenes*, *Campylobacter* spp. and STEC regardless of sample type. *Loose housed herds
